# Middle Meningeal Artery Embolization as a First-Line Therapy for Chronic Subdural Hematoma

**DOI:** 10.7759/cureus.99662

**Published:** 2025-12-19

**Authors:** Rahul Jain, Armaan Shah, David Mina, Andre Beer Furlan, Mustafa Al-Roubaie

**Affiliations:** 1 Interventional Radiology, University of South Florida Health Morsani College of Medicine, Tampa, USA; 2 Radiology, Northwell Health, New York, USA; 3 Interventional Radiology, Moffitt Cancer Center, Tampa, USA; 4 Neurosurgery, Moffitt Cancer Center, Tampa, USA

**Keywords:** chronic subdural hematoma (csdh), first-line treatment, leptomeningeal metastasis (lm), middle meningeal artery embolization, percutaneous endovascular therapy

## Abstract

Chronic subdural hematoma (cSDH) is a common neurovascular condition, particularly among elderly or anticoagulated patients. Its recurrence after surgical evacuation remains a major challenge. Middle meningeal artery embolization (MMAE) has recently emerged as a minimally invasive and durable alternative treatment, targeting the vascular networks that sustain hematoma membranes.

We describe a patient with metastatic prostate cancer who developed a spontaneous cSDH and underwent first-line MMAE due to high surgical risk. Angiography revealed multifocal patchy dural enhancement resembling tumor blush, which was later confirmed on MRI to represent leptomeningeal metastasis. To our knowledge, the middle meningeal angiographic appearance of leptomeningeal disease seen in this case is previously unpublished. Embolization using 100-300 μm embospheres and coils achieved complete occlusion of the vessel and clinical improvement without recurrence.

This case highlights the growing role of MMAE as a safe, effective first-line therapy for cSDH, supported by recent clinical trials demonstrating significant reductions in recurrence compared with conventional management. This report also underscores the importance of correlating angiographic findings with MRI when unique neoplastic vascular patterns are suspected.

## Introduction

Chronic subdural hematoma (cSDH) is a prevalent neurovascular condition, especially among older adults and patients on antithrombotic therapy, and its incidence is projected to rise [1]. The pathogenesis is driven by the formation of fragile neovasculature within the subdural membrane, predominantly supplied by branches of the middle meningeal artery (MMA). This vascular network perpetuates a cycle of chronic inflammation, recurrent micro-hemorrhages, and fluid exudation, leading to hematoma expansion [2-5]. While traditional surgical evacuation provides rapid decompression, it does not address this underlying vascular source, and recurrence remains a significant clinical challenge.

MMA embolization (MMAE) has emerged as a minimally invasive therapeutic strategy that directly targets the pathophysiology of cSDH by occluding this neovascular supply. Recent meta-analyses and randomized controlled trials have demonstrated that MMAE, either as an adjunct to surgery or as a standalone therapy, significantly reduces recurrence rates compared to conventional management, without increasing morbidity or mortality [6-9]. This case report describes the successful use of first-line MMAE in a high-risk patient with metastatic prostate cancer who developed a spontaneous cSDH. We highlight a previously undescribed angiographic finding of patchy dural enhancement resembling "tumor blush," which was later confirmed on MRI to represent leptomeningeal metastasis, underscoring the importance of correlating imaging findings in this complex patient population [10-13].

## Case presentation

**Figure 1:**
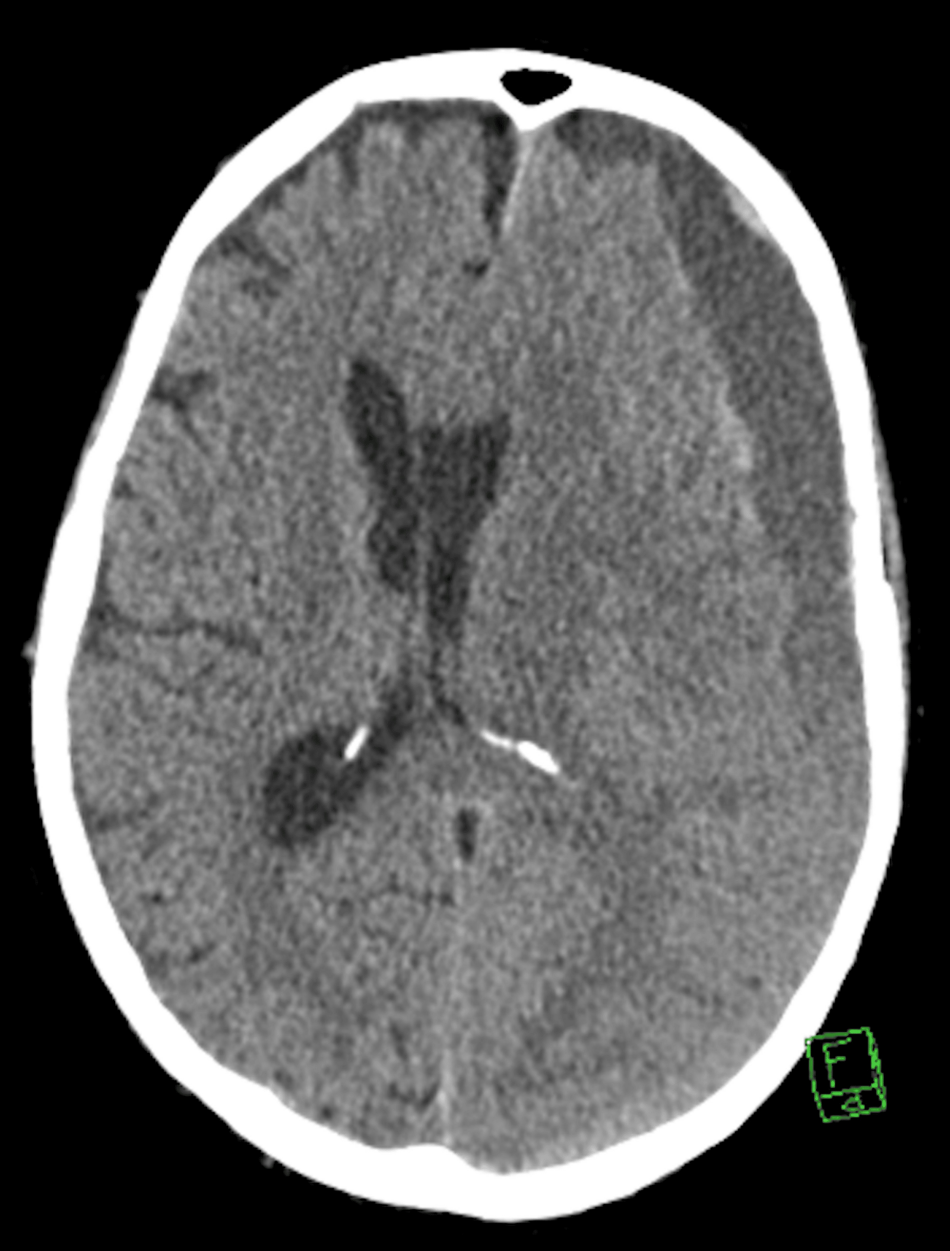
Non-contrast head CT showing a large left-sided subdural hematoma with mixed densities suggesting an acute-on-chronic hemorrhage. The hematoma measured 2.0 cm in greatest depth with 1.0 cm of rightward midline shift.

**Figure 2:**
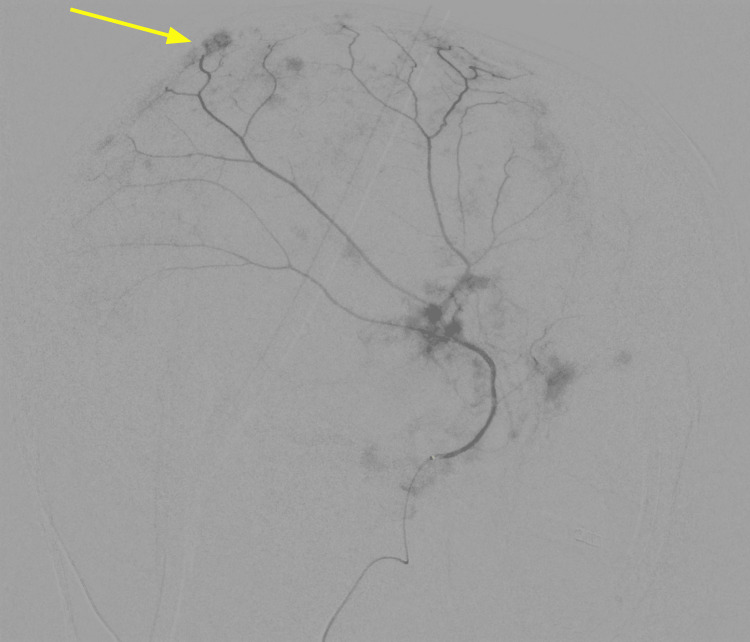
Left MMA angiogram demonstrates multiple patchy areas of 'tumor blush' (arrow) in the dural branches, an unusual finding later confirmed to represent neoplastic vascularity from leptomeningeal disease. MMA: middle meningeal artery

**Figure 3:**
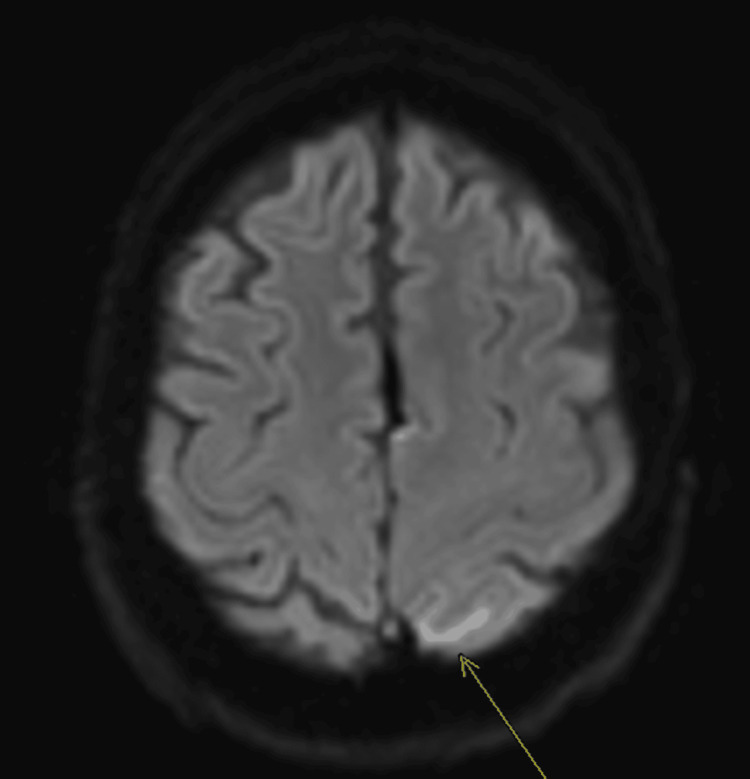
Axial T2 FLAIR MRI showing patchy areas of pial hyperintensity in the left subparietal region (arrow). These findings correlate anatomically with the angiographic 'tumor blush' seen in Figure 2, confirming leptomeningeal metastatic involvement. FLAIR: fluid attenuated inversion recovery

A 74-year-old male with prostate cancer metastatic to the lungs on peptide receptor radionuclide therapy presented with acute altered mental status. His spouse reported a new onset of lethargy and confusion for about one day. He was not alert or oriented by physical exam and had no focal neurological deficits. CT imaging found a large acute left subdural hematoma (Figure 1). There was no basal cistern effacement to suggest brain herniation. The patient was observed and showed clinical improvement over three days. During this time, he remained hemodynamically stable, and while he had an altered mental status, he did not exhibit any neurological deficits. Neurosurgery consultation was obtained, and the recommendation was empiric MMAE to prevent any SDH recurrence. During diagnostic angiography, the left MMA demonstrated patchy areas of enhancement involving the dural branches, resembling areas of “tumor blush” (Figure 2). Subsequent T2 fluid-attenuated inversion recovery (FLAIR) MRI imaging confirmed this finding to represent leptomeningeal metastasis in the left subparietal region (Figure 3). Embolization of the three left MMA branches was performed with 100-300 μm particles, and one 2 mm x 6 cm coil deployed at the distal left MMA trunk, distal to the petrous branches, resulting in complete devascularization of the abnormal vascular territory. No orbital or lacrimal branches were identified. The patient tolerated the procedure without complication and continued to recover in the hospital. A two-week follow-up with neurosurgery showed resolution of altered mental status, and a repeat head CT showed a reduction in the size of the hematoma.

## Discussion

MMAE has rapidly evolved into a cornerstone therapy for cSDH, offering a less invasive and more durable alternative to conventional surgical evacuation. The rationale is compelling: by occluding the distal MMA branches that feed the hematoma’s vascularized outer membrane, MMAE directly targets the source of the chronic bleeding and fluid exudation that drives hematoma persistence and recurrence [4,5]. A robust body of evidence from multiple meta-analyses and randomized controlled trials now confirms that MMAE, whether used as an adjunct to surgery or as a standalone therapy, significantly reduces the rates of hematoma recurrence and the need for surgical rescue when compared to conventional management alone, all without increasing major complications or mortality [1,3,6-8].

The presented case contributes to this growing evidence base but is differentiated by several unique clinical and radiographic features. We report the successful use of MMAE as a first-line therapy for a spontaneous cSDH in a patient with active metastatic cancer and high surgical risk [10-13]. Critically, this case also features a rare angiographic finding, patchy dural enhancement resembling "tumor blush," which was subsequently correlated with MRI findings of leptomeningeal metastasis.

This angiographic appearance is a particularly noteworthy finding and presents a potential diagnostic pitfall. While the typical angiographic sign of cSDH is a persistent, homogenous "cotton-wool"-like dural enhancement, the patchy, blush-like pattern seen in our patient is highly atypical. The differential diagnosis for such a finding could include a dural arteriovenous fistula, hypervascular meningioma, or a primary inflammatory process. To our knowledge, the specific angiographic visualization of leptomeningeal metastasis via MMA injection in the setting of a cSDH has not been previously detailed in the literature. This finding underscores the importance of maintaining a high index of suspicion for underlying pathology in cases of spontaneous cSDH, especially in oncologic patients. It also demonstrates the critical need to correlate ambiguous or unusual angiographic findings with high-resolution, cross-sectional imaging like MRI to arrive at a definitive diagnosis and avoid misinterpretation.

Furthermore, the decision to use MMAE as the primary treatment strategy in this context is a key aspect of this case. In the largest available series and reviews, MMAE in cancer patients is typically described for hematomas that are refractory to surgery or in the setting of severe coagulopathy or thrombocytopenia [7,11]. In contrast, our patient received MMAE as the intended first-line treatment due to general surgical risk, demonstrating its utility earlier in the treatment paradigm for this complex population. The successful outcome, resolution of symptoms, reduction in hematoma size, and no recurrence highlight the therapeutic potential of MMAE to provide a definitive treatment that can facilitate a quicker return to systemic cancer therapy, a crucial goal in oncologic care.

The procedure itself was safe and effective, achieving complete devascularization of the abnormal territory using standard embolic particles and a coil, with no complications. Functional outcomes were excellent, with the patient returning to his neurological baseline. While different embolic agents like liquid embolics have been studied, our case supports the efficacy of particles and coils in achieving excellent clinical and radiological outcomes [4,5]. This reinforces that MMAE is not only a viable alternative to surgery but, in select high-risk patients, may be the preferable initial approach to manage cSDH and its underlying drivers [13-17].

## Conclusions

This case highlights the expanding role of MMAE as a safe and effective treatment option for chronic subdural hematoma, particularly in patients for whom surgery carries an elevated risk. By targeting the neovascular supply driving hematoma persistence, MMAE directly addresses the underlying pathophysiology and has been shown to significantly reduce recurrence rates compared with conventional management. In this patient, first-line embolization resulted in complete devascularization and clinical stability without recurrence. The additional finding of a leptomeningeal “tumor blush” underscores the importance of careful angiographic interpretation and correlation with MRI to avoid diagnostic pitfalls. This case supports MMAE not only as an adjunct but also as a potential first-line therapy in appropriately selected patients, particularly those with high surgical risk or complex comorbidities such as active malignancy.
